# Readmission rates in not-for-profit vs. proprietary hospitals before and after the hospital readmission reduction program implementation

**DOI:** 10.1186/s12913-018-2840-4

**Published:** 2018-01-19

**Authors:** Lauren E. Birmingham, Willie H. Oglesby

**Affiliations:** 1 Kent State University, College of Public Health, PO Box 5190, Kent, OH 44242 USA; 2 Thomas Jefferson University, College of Population Health, 901 Walnut Street, 10th Floor, Philadelphia, PA 19107 USA

**Keywords:** Readmissions, Healthcare quality, Hospital, Not-for-profit hospital, Proprietary hospital, Health policy

## Abstract

**Background:**

The Patient Protection and Affordable Care Act established the Hospital Readmission Reduction Program (HRRP) to penalize hospitals with excessive 30-day hospital readmissions of Medicare enrollees for specific conditions. This policy was aimed at increasing the quality of care delivered to patients and decreasing the amount of money paid for potentially preventable hospital readmissions. While it has been established that the number of 30-day hospital readmissions decreased after program implementation, it is unknown whether this effect occurred equally between not-for-profit and proprietary hospitals. The aim of this study was to determine whether or not the HRRP decreased readmission rates equally between not-for-profit and proprietary hospitals between 2010 and 2012.

**Methods:**

Data on readmissions came from the Dartmouth Atlas and hospital ownership data came from the Centers for Medicare and Medicaid Services. Data were joined using the Medicare provider number. Using a difference-in-differences approach, bivariate and regression analyses were conducted to compare readmission rates between not-for-profit and proprietary hospitals between 2010 and 2012 and were adjusted for hospital characteristics.

**Results:**

In 2010, prior to program implementation, unadjusted readmission rates for proprietary and not-for-profit hospitals were 16.16% and 15.78%, respectively. In 2012, following program implementation, 30-day readmission rates dropped to 15.76% and 15.29% for proprietary and not-for-profit hospitals. The data suggest that the implementation of the Hospital Readmission Reduction Program had similar effects on not-for-profit and proprietary hospitals with respect to readmission rates, even after adjusting for confounders.

**Conclusions:**

Although not-for-profit hospitals had lower 30-day readmission rates than proprietary hospitals in both 2010 and 2012, they both decreased after the implementation of the HRRP and the decreases were not statistically significantly different. Thus, this study suggests that the Hospital Readmission Reduction Program was equally effective in reducing readmission rates, despite ownership status.

## Background

In the United States, the ownership status of a hospital can take many forms. According to data from the Centers for Medicaid and Medicare Services (CMS), there are 11 distinct hospital ownership categories, including various types of government and not-for-profit hospitals, as well as proprietary, physician-owned, and tribal hospitals. According to 2014 data from CMS, which includes only hospitals that bill CMS, approximately 1200 hospitals were government-owned, nearly 3000 were not-for-profit, and about 800 were proprietary (or for-profit).

There are numerous differences between not-for-profit and proprietary hospitals. The primary difference is that not-for-profit hospitals are not subject to certain state or federal taxes, but in return must report on the amount of community benefit they perform in the community in which the hospital resides [1]. For-profit hospitals can raise capital through investors, which may help these institutions access high-tech equipment, which has been hypothesized to increase quality outcomes [1]. However, for-profit hospitals are responsible to shareholders, whose interests may or may not be aligned with patient interests, causing some to argue that for-profit hospitals do not always have a “patients first” mentality [1].

Other major differences between not-for-profit and proprietary hospitals are their financial health and forecasts for growth. In reports from 2008 and 2013, Moody’s Investor Service has had a negative outlook on US-based not-for-profit hospitals [2, 3]. This is due to growing costs and decreasing revenues from payers. Reimbursements have declined over time, making margins tighter. Decreased demand for health care services, namely through weak employment rates, reduced reimbursement from government payers [2], and increases in retail clinic usage and high-deductible health insurance plans have had negative impacts on utilization [4]. For-profit hospitals may have been somewhat shielded from this, as they are able to raise revenue through other means are able to control costs by not providing care for low-reimbursing patients and by not providing low margin services. As a result, not-for-profit hospital costs have grown at a higher rate than revenues resulting in a net operating loss for approximately 25% of not-for-profit hospitals in 2013 [4]. A study examining the economic impact of converting from a not-for-profit to a for-profit hospital found that after such conversions, the hospitals’ total margins improved, on average, from 0.4% to 2.2% [5].

The Patient Protection and Affordable Care Act of 2010 (ACA) established many policies to improve patient outcomes and reduce health care expenditures. One of these policies was the 30-day readmission penalty, also known as the Hospital Readmission Reduction Program (HRRP). The HRRP became effective on October 1, 2012 and penalized hospitals with excessive Medicare beneficiary readmissions for select conditions (heart failure, heart attack, and pneumonia in 2012) with a maximum fee of 1% reduction in Medicare reimbursements. In 2012, the first year of implementation, 307 hospitals faced the maximum penalty, resulting in $280 million dollars in loss to the hospitals with excessive readmissions for these conditions [6]. Although some hospitals were penalized, research has shown that this program has been effective in reducing readmission rates overall [7]. However, the effect of hospital characteristics on this overall reduction in readmission rates is not as well-understood. Thus, the purpose of this analysis was to determine if implementation of the HRRP impacted readmission rates differently between not-for-profit and proprietary hospitals.

Not-for-profit and proprietary hospitals have documented differences in clinical quality and patient outcomes. This is a difficult topic to study as there are many confounders and other factors that drive differences in quality outside of the ownership model. McClelland and Staiger (2000) were able to overcome some of these difficulties and showed that non-profit hospitals had lower mortality rates when compared to proprietary hospitals [8]. Furthermore, broader evaluations of hospital quality, outside of mortality, have also demonstrated differences between not-for-profit and proprietary hospitals, with not-for-profit hospitals typically performing better than proprietary hospitals [9, 10].

Theoretically, given that not-for-profit hospitals already have relatively weaker financial performance, on average, than their proprietary counterparts [11], a 1% reduction in Medicare payments could have induced a greater incentive to improve care with respect to readmissions among not-for-profit hospitals than proprietary hospitals. Thus, the primary aim of this study was to determine if the rate of change in readmission rates before and after implementation of the HRRP was the same for not-for-profit and proprietary hospitals. It has been shown that not-for-profit hospitals are willing to change their practices to address market conditions that threaten their viability [12, 13], so it is not unreasonable to believe that not-for-profit hospitals might strategically react to the HRRP with more urgency than proprietary hospitals; especially given the tighter operating margins of not-for-profit hospitals.

The first research question asks if readmission rates at baseline (2010) and the post-ACA period (2012) are different for proprietary and not-for-profit hospitals. The second research question asks if the readmission rates decreased between 2010 and 2012 for both types of hospitals. Lastly, the third research question asks if the rates declined at different magnitudes. It is hypothesized that the HRRP policy caused readmission rates to decline for both not-for-profit and proprietary hospitals, but that they declined more in not-for-profit hospitals where the penalty would be more difficult for the hospital to absorb. These are important questions to answer in order to assess the overall effectiveness of the HRRP in reducing readmissions and to understand if the penalty incentivized better performance relative to readmissions equally in not-for-profit and for-profit hospitals. As the rate of hospital consolidation increases [14–16], and financially weak hospitals are acquired by stronger hospitals with varying ownership statuses, the effect of ownership status on quality needs to be understood.

## Methods

This was a quasi-experimental study that utilized a controlled before-and-after design as described elsewhere [17]. This study compares the differences in readmission rates from 2010 to 2012 between proprietary and not-for-profit hospitals. This study was found to involve minimal risk to human subjects because it used publically available existing data, and was granted exemption by the Kent State University Institutional Review Board.

### Availability of data and materials

**Fig. 1:**
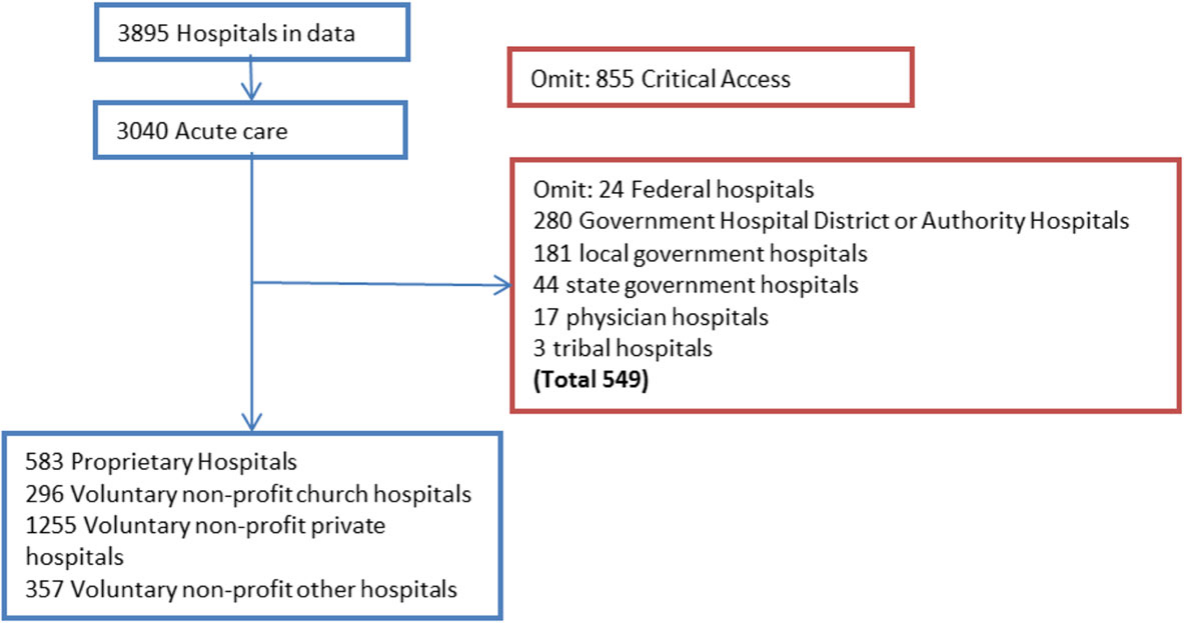
Hospital Flow Diagram

Data on hospital-specific 30-day readmission rates from 2010 and 2012 were gathered from publically available data in the Dartmouth Atlas of Healthcare, which aggregates data from the CMS Hospital Compare website [18]. Data on Maryland hospitals is suppressed by the Dartmouth Atlas because Maryland hospitals report readmissions differently than other states, making the comparison imbalanced. Data on hospital ownership status, hospital characteristics, and case mix indices were taken directly from the CMS Hospital Compare web site. Median income data by zip code were taken from the 2006-2010 American Community Survey, which is publicly available from the University of Michigan Population Studies Center. Data were joined together using the Medicare provider number. The hospital flow diagram depicted in Fig. 1 summarizes how the analytic dataset was constructed.

### Outcome and exposure variables

The 30-day all-cause Medicare readmission rate was the primary outcome measure in this analysis. The 30-day readmission rate was calculated by CMS using a methodology that has been described elsewhere [19]. This variable was available for each hospital for both time periods (2010 and 2012) and is a 30-day all-cause readmission for Medicare patients who made an initial visit for a medical (not surgical) reason.

The primary exposure was hospital ownership type, which could only take two values in this study: not-for-profit or proprietary. All other ownership types were excluded from this analysis (e.g., government hospitals, tribal hospitals, etc.).

### Covariates

Case mix index (CMI) is often used as a proxy for illness severity and when aggregated for an entire hospital, it can provide insight into average case severity at a hospital [20]. CMI was included as a covariate for both 2010 and 2012 since a hospital’s average patient illness severity could affect its readmission rates. Median family income for the zip code in which the hospital resides was included as a proxy for the socioeconomic status of the community the hospital serves, given the relationship between socioeconomic status and negative health outcomes. A binary variable representing whether or not a hospital had an emergency department (ED) was included because some literature has discussed the potential for patients to be boarded in the ED to avoid readmission [21, 22].

### Statistical analysis

The first research question asks if readmission rates differed significantly by ownership status in 2010 and 2012 separately. This was first examined by simple independent t-tests to detect differences in means. Then, regression analysis was used to model the 2010 and 2012 readmission rates separately, while controlling for other factors. The second research question asks if the readmission rate decreased from 2010 to 2012. This question is first explored using a paired t-test. Repeated measures multiple linear regression analysis is subsequently used to model the difference in readmission rates between 2010 and 2012. This statistical method was also used to answer the final research question regarding the potential for differences in the rate of change in readmission rates between 2010 and 2012 for not-for-profit and proprietary hospitals, utilizing a difference-in-differences term. A *p* < 0.05 level of significance (alpha) was used in all analyses. All statistical analyses were conducted in SAS 9.3 (Cary, NC).

## Results

Table 1 reports the results of the bivariate analysis. The 2010 readmission rates are significantly different based on hospital ownership (proprietary 16.16% and not-for-profit 15.78%). In 2012, the readmission rates decreased in both ownership categories and remained significantly different from one another*.* On average, the readmission rate for proprietary hospitals decreased by 0.40% and the not-for-profit readmission rate decreased by 0.49%. This data also demonstrates differences in other factors by ownership status. CMI in 2010 and 2012 was significantly higher in not-for-profit hospitals than proprietary hospitals and both hospital types observed absolute increases in CMI between 2010 and 2012.

**Table 1:** Population Characteristics

	Hospital Ownership				
	Proprietary *n* = 583		Not-for-profit *n* = 1908		
	Mean	Std Dev	Mean	Std Dev	*p*-value
Percent 30-day readmissions 2010	16.16%	2.04	15.78%	2.35	*0.0003* ^*a*^
Percent 30-day readmissions 2012	15.76%	2.15	15.29%	2.20	*< 0.0001* ^*a*^
Median Income in hospital zip code	$64,222	$25,122	$66,879	$28,155	*0.0343* ^*a*^
Case Mix Index (2010)	1.40	0.29	1.45	0.26	*0.0016* ^*a*^
Case Mix Index (2012)	1.44	0.29	1.49	0.26	*0.0011* ^*a*^
	%	n	%	n	
No emergency department at hospital	3.77%	22	2.41%	46	0.1153

*Note:* ^*a*^ *italics represents statistically significant findings*

Hospital readmission rates decreased significantly between 2010 and 2012 by 0.46% on average (95% CI 0.37, 0.55). When stratified by ownership status, proprietary hospitals and not-for-profit hospitals both had statistically significant decreases in readmission rates (*p* = 0.0002, *p* < 0.0001, respectively). Case mix index increased between 2010 and 2012 for both ownership types and is higher in not-for-profit hospitals than proprietary hospitals. The difference in rates of hospitals without emergency departments was not significantly different between proprietary and not-for-profit hospitals.

While the results of the bivariate analysis provided insight into the first two research questions, this method does not control for other factors, such as case severity treated at the respective hospitals, or neighborhood median income (a proxy for socioeconomic status). Two regression models were built to model readmission rates in 2010 and 2012 and included a dummy variable indicating either not-for-profit or proprietary status, the associated year’s case mix index (CMI), presence of an emergency department at the hospital, and the natural logarithm of the median income of the neighborhood in which the hospital resides. Results are summarized in Table 2.

**Table 2:** Modeled Average Readmission Rates for 2010 and 2012 Not-for-Profit and Proprietary Hospitals

	2010 Model Estimate (95%CI)	2012 Model Estimate (95%CI)
Intercept	21.87 (18.98, 24.76)*	22.19 (19.36, 25.02)*
Not-for-profit status	−0.44 (− 0.66,-0.23)*	− 0.51 (− 0.72,-0.31)*
CMI	0.54 (0.18, 0.89)*	0.19 (− 0.16, 0.54)
Log Median Income	− 0.58 (− 0.84, − 0.32)*	− 0.65 (− 0.91, − 0.40)*
Presence of Emergency Department	−0.03 (− 0.72, 0.65)	0.50 (− 0.17, 1.18)

*The asterisk denotes statistically significant estimates at the alpha = 0.05 level

Table 2 shows that the average effect of not-for-profit status on readmission rates in both 2010 and 2012, controlling for CMI, median income of the hospital area, and presence of a hospital-based ED. While the point estimate for not-for-profit status is lower in the 2012 readmission rate model compared to the 2010 model, the confidence intervals overlap, which indicates that they are not significantly different from one another. Thus, while the point estimate is lower (meaning, on average, it is expected that a not-for-profit hospital would have a lower readmission rate than a proprietary hospital, all other things equal), the difference is not statistically significant. The log of median income is significant in both models, indicating income is negatively associated with readmission rates. Case mix index was only significant in the 2010 model.

Table 3 presents the results of unadjusted simple linear regression modeling the change in readmission rate between 2010 and 2012. This data demonstrates that no single variable was statistically significantly associated with the average change in readmission rate from 2010 to 2012, including the not-for-profit status variable. This suggests that the change in readmission rate from 2010 to 2012 did not differ by ownership status, but adjusted models with a difference-in-difference term were created to further investigate this.

**Table 3:** Change in Readmission Rates from 2010 to 2012: Simple Linear Regression

	Unadjusted Simple Linear Regression (95% CI)
Not-for-profit status	−0.08 (− 0.29, 0.14)
CMI 2010	− 0.29 (− 0.64, 0.06)
CMI 2012	−0.22 (− 0.57, 0.12)
Presence of Emergency Department	0.47 (− 0.21, 1.16)
Log Median Income	−0.11 (− 0.37, 0.15)

Difference-in-differences models were evaluated to determine if a multivariable approach would demonstrate any differences between not-for-profit and proprietary hospitals with respect to the change in readmission rates between 2010 and 2012. The first model (DiD Model 1) is a simple model, including only the DiD term and its components. The DiD term was not significant, suggesting there was no change in the rate at which not-for-profit and proprietary hospitals experienced decreased readmission rates, which is not surprising given the findings in Table 2. DiD models 2-4 included additional terms including the natural log of median income, CMI 2010 (CMI 2012 was also evaluated and found to not be statistically significant) and the lack of an emergency department at the hospital. Median income and the CMI in 2010 were significant singularly in the models (DiD Model 2 and 3, respectively) as well as in the final model (DiD Model 5). The impact of having an emergency department on readmission rates was also evaluated (DiD Model 4). This factor was significant in DiD Model 4, but lost statistical significance in DiD Model 5 (Table 4).

**Table 4:** Difference-in-Differences Models

	DiD Model 1 Parameter Estimate (SE)	DiD Model 2 Parameter Estimate (SE)	DiD Model 3 Parameter Estimate (SE)	DiD Model 4 Parameter Estimate (SE)	DiD Model 5 Parameter Estimate (SE)
Intercept	16.16 (0.10)*	22.68 (1.20)*	15.64 (0.22)*	17.57 (0.29)*	22.72 (1.21)*
Not-for-profit Indicator	−0.31 (0.11)*	− 0.42 (0.10)*	−0.44 (0.10)*	− 0.38 (0.11)*	−0.44 (0.11)*
Year 2012 Indicator	−0.38 (0.10)*	−0.39 (0.10)*	− 0.40 (0.10)*	−0.38 (0.09)*	− 0.41 (0.10)*
Not-for-profit * Year 2012 (DiD term)	−0.10 (0.11) n.s.	− 0.10 (0.11) n.s.	−0.08 (0.11) n.s.	− 0.10 (0.11) n.s.	−0.08 (0.11) n.s.
Log Median Income		−0.59 (0.11)*			−0.65 (0.11)*
CMI 2010			0.37 (0.14)*		0.45 (0.15)*
Emergency Department				−1.44 (0.29)*	−0.07 (0.29) n.s.
AICC	19,704	18,250	19,081	19,679	18,037
BIC	19,715	18,265	19,092	19,690	18,049

*The asterisk denotes statistically significant estimates at the alpha = 0.05 level

## Discussion

The results of this analysis suggest that, on average, not-for-profit hospitals had lower 30-day readmission rates than proprietary hospitals in both 2010 and 2012, even after adjustment. This is consistent with previous findings on 2006-2007 hospital readmission rates, where not-for-profit hospitals were found to have more favorable readmission rates [23]. Despite differences at baseline, this analysis found that readmission rates decreased for both not-for-profit and proprietary hospitals between 2010 and 2012, on average. This is also in agreement with previous literature finding a decline in overall hospital CMS-calculated readmission rates in similar time periods [7, 21, 24, 25]. Most recently, Zuckerman et al. (2016) reported that the readmission rates for conditions targeted by the HRRP decreased by 3.7 percentage points from 2007 to 2015 [7]. Similarly, an analysis found a 0.3% decrease in all-condition readmission rates between 2009 and 2011 in a national study using Medicare data [25]. A different national analysis of Medicare data found a 1% decrease in all-cause readmission rates from 2007 to 2012 [24].

The central research question of this study asked if the readmission rates declined at the same rate from 2010 to 2012 for both not-for-profit and proprietary hospitals. The difference-in-differences methodology provided a means to answer this question with more sophistication than a simple comparison of rates. These data demonstrate that rates did not decline at significantly at different rates, suggesting the HRRP policy implementation was equally effective in reducing readmissions between not-for-profit and proprietary hospitals. Data did not support the original hypothesis that not-for-profit hospitals may have found the prospect of the HRRP penalties to be more threatening given their relatively weaker financial positions, compared to their for-profit counterparts, and would reduce readmission rates more aggressively. Previous studies of hospital quality have shown that not-for-profit hospitals perform better on quality metrics than proprietary hospitals [8–10, 26]. For this reason, it is important to assess how health policies differentially impact hospitals and patients by ownership status so as to not widen this already present disparity.

One concept that has been discussed with respect to readmission reductions is boarding patients in the emergency department to avoid an inpatient admission [7]. Our analysis does not support this theory which is consistent with other literature [7], although it was not a direct focus of the analysis. Only 45 hospitals in the sample did not have emergency departments in both 2010 and 2012, making it difficult to detect significant small changes in readmissions rates. The binary emergency department indicator variable was not significantly associated with changes in the rates of readmission after the implementation of the HRRP in the final adjusted DiD model, nor was it significant in the 2010 and 2012 readmission rate models. The issue of boarding patients in the emergency department as a means to prevent readmissions is certainly not settled by this analysis, but this theory is not supported by this data.

This analysis is not without weaknesses. The primary weakness of the regression analysis is the likelihood of omitted variables. Ideally more information on patient factors associated with hospital readmission would be included. Median income and case mix index were added to attempt to control for this effect, but these variables are not a perfect proxy for individual characteristics that explain variation in readmissions. Additionally, more information on other hospital characteristics that could be associated with hospital quality or readmissions would ideally be included. This could include participation in quality networks, the presence of an observation unit (where patients could theoretically be boarded), teaching status, etc. Future research could control for known omitted variables through a matched study design, which could potentially reduce the risk of bias.

The primary assumption made in difference-in-difference analyses is the parallel trends assumption, indicating that, without the intervention (e.g., the HRRP), one would expect not-for-profit and proprietary hospitals to have parallel readmission rate trends overtime. Prior to ACA, in 2008 and 2009, readmission rates changed at similar rates for not-for-profit and proprietary hospitals. The average readmissions rate for not-for-profit hospitals was 15.6% in both 2008 and 2009, and 16.1% and 16.0% for proprietary hospitals. Given the consistency in readmission rates between the 2 years, this provides some evidence in support of the parallel trends assumption, although examining data prior to this time period could provide greater insight, in addition to adjustment for patient characteristics that can impact the readmission rate.

Data limitations existed, notably the requirement for each hospital to have a readmission rate for both 2010 and 2012, thus hospitals that existed in 2012 but not in 2010 (and vice versa) were not able to be included in the analysis. As previously mentioned, the analysis of hospital-based emergency department suffered from a small sample size, since so few hospitals do not have emergency rooms (68 hospitals in total, and only 45 in both 2010 and 2012). Another potential problem was the potential for misclassification. Some hospitals may be classified as not-for-profit hospitals, but may operate like a for-profit hospital (or vice versa) in terms of their financial motivations, adding heterogeneity to the group. While legal requirements exist to prevent this practice, it may still be the case that some not-for-profits have more in common with for-profit institutions than not-for-profit institutions. Further misclassification may have occurred, since the CMS data used to generate the not-for-profit vs. proprietary designation was from 2014, and the readmissions data was from 2010 and 2012. Over this time period, hospitals may have changed ownership status. While it is not possible to measure the prevalence with which this occurred with the current dataset, the likelihood of this occurring may be relatively small, since this would require a hospital to switch to a not-for-profit or proprietary ownership status between 2010 and 2012 without also changing Medicare Provider Number, as often occurs during acquisitions.

A strength of this analysis is the relatively large sample size of hospitals. There were 1908 not-for-profit and 583 proprietary hospitals included in the analysis. This was a nationwide analysis, and as such, was not limited to one geographic part of the United States or one hospital or health system. The results of this study with respect to readmission reductions over time are largely in agreement with previous literature [23–25], although this analysis provides an updated analysis, post-Affordable Care Act implementation. To the best of our knowledge, no other study has directly assessed the effect of the HRRP on readmission rates by ownership status overtime, making this a unique contribution.

## Conclusion

This study found that readmission rates differed in absolute terms, with not-for-profit hospitals having lower rates of readmission than proprietary hospitals. Both not-for-profit and proprietary hospitals experienced decreases in readmission rates from 2010 to 2012, but the rate of decline was not different even after adjustments for commonly suspected confounders were made. This analysis supports the claim that the Hospital Readmission Reduction Program impacted readmissions rates with similar effectiveness for both not-for-profit and proprietary hospitals. As new health policies are developed in the future, the effectiveness of this policy should be taken into account as it has been associated with declines in readmission rates across hospital ownership models, and has not widened known quality disparities by ownership status. More advanced methodology may improve the accuracy of these results by using propensity score matching, or another method that reduces the impact of non-random selection into the primary exposure group.

The relationship between quality and hospital ownership should continue to be examined, and evolve into questions about the relationship between quality and market position or market concentration. Evidence suggests that hospitals in monopolistic markets have poorer quality outcomes [27], thus assessing the impact of the HRRP on monopolistic vs. competitive markets could provide insight into the effectiveness of this policy in markets where a hospital does not need to compete on quality metrics to attract customers. These analyses will help policy-makers understand how policy implementation impacts the targeted problem, and will provide insight into how to improve policy development in the future. This is imperative as federal and state health policy efforts continue to focus on controlling health care costs and improving quality.

## Abbreviations

ACA: Affordable Care Act
ACS: American Community Survey
CMI: Case mix index
CMS: Center for Medicare and Medicaid Services
HRRP: Hospital Readmission Reduction Program
